# Rectal swabs as a viable alternative to faecal sampling for the analysis of gut microbiota functionality and composition

**DOI:** 10.1038/s41598-022-27131-9

**Published:** 2023-01-10

**Authors:** Shiva T. Radhakrishnan, Kate I. Gallagher, Benjamin H. Mullish, Jose I. Serrano-Contreras, James L. Alexander, Jesus Miguens Blanco, Nathan P. Danckert, Maria Valdivia-Garcia, Billy J. Hopkins, Anik Ghai, Azad Ayub, Jia V. Li, Julian R. Marchesi, Horace R. T. Williams

**Affiliations:** 1 Departments of Gastroenterology and Hepatology, St Mary’s Hospital, Imperial College Healthcare NHS Trust, London, UK; 2 Division of Digestive Diseases, Department of Metabolism, Digestion and Reproduction, Faculty of Medicine, Imperial College London, London, UK; 3 Department of Twins Research and Genetic Epidemiology, Faculty of Life Sciences & Medicine, King’s College London, London, UK; 4 Department of Acute Medicine, University College London Hospitals NHS Foundation Trust, London, UK

**Keywords:** Gastrointestinal diseases, Gastrointestinal system, Microbiome, Translational research

## Abstract

Faecal or biopsy samples are frequently used to analyse the gut microbiota, but issues remain with the provision and collection of such samples. Rectal swabs are widely-utilised in clinical practice and previous data demonstrate their potential role in microbiota analyses; however, studies to date have been heterogenous, and there are a particular lack of data concerning the utility of swabs for the analysis of the microbiota’s functionality and metabolome. We compared paired stool and rectal swab samples from healthy individuals to investigate whether rectal swabs are a reliable proxy for faecal sampling. There were no significant differences in key alpha and beta diversity measures between swab and faecal samples, and inter-subject variability was preserved. Additionally, no significant differences were demonstrated in abundance of major annotated phyla. Inferred gut functionality using Tax4Fun2 showed excellent correlation between the two sampling techniques (Pearson’s coefficient r = 0.9217, *P* < 0.0001). Proton nuclear magnetic resonance (^1^H NMR) spectroscopy enabled the detection of 20 metabolites, with overall excellent correlation identified between rectal swab and faecal samples for levels all metabolites collectively, although more variable degrees of association between swab and stool for levels of individual metabolites. These data support the utility of rectal swabs in both compositional and functional analyses of the gut microbiota.

## Introduction

At present, analysis of the gut microbiota in humans primarily necessitates provision of a faecal sample or a mucosal biopsy. Current methods of faecal sampling are not without drawbacks; in particular, the very nature of defecation means that samples cannot be provided ‘on demand’ in a physician’s office or to a research nurse and are reliant on appropriate collection by patients themselves. Faeces sampling may also present additional complexities, including the logistical challenges of having to transport samples between patient’s home, the clinic and the laboratory, often with careful attention to maintaining cold chain conditions in the process^1^. Qualitative research demonstrates that patients are reluctant to handle their own stool and are embarrassed about transporting faeces^2^. Such aversion to traditional methods is mirrored in an inflammatory bowel disease (IBD) population, where compliance with stool collection to obtain faecal calprotectin measurements may be as low as 35%^3^. An alternative option to faecal sampling is colonic biopsy sampling at the time of an endoscopic procedure. In addition to the requirement for an invasive examination, endoscopic sampling frequently requires bowel preparation, which is well-recognised to affect intestinal microbiota composition^4^. As such, other options for obtaining samples to assess the human distal gut microbiota are of key interest.

Rectal swabs are widely-used in clinical practice; for example, national U.K guidance mandates their use for screening for intestinal colonization with carbapenemase-producing *Enterobacteriaceae* (CPE) for at-risk patients admitted to healthcare settings^5^. Studies of patient opinions demonstrate high levels of acceptability for CPE detection and rectal swabbing as a method of sample collection^6^. Given their acceptability, ease of administration, ease of self-administration and existing utility in clinical microbiology, rectal swabs might represent an attractive means for sampling the broader gut microbiota and aspects of the gut metabolome. However, the degree to which rectal swabs and faecal samples offer comparable assessment of the microbiota remains uncertain. Pilot studies have demonstrated close correlation between gut bacterial composition and alpha diversity in rectal swabs and matched faecal samples in both adult and pediatric populations^7,8^. An area of growing interest in this field relates to extending beyond profiling gut microbiota composition alone to also explore gut microbiota functionality; in particular, such “multi-omic” analyses are advocated to better delineate the interplay between microbe and human host^9^. One such key ‘omics’ technology is metabolomics, whereby advanced analytical chemistry techniques (including nuclear magnetic resonance spectroscopy (NMR) and mass spectroscopy (MS)) are used to identify and quantify small molecules within biofluids. While comparison of swabs *versus* stool profiles has already been investigated on a small scale for certain defined metabolite groups (including bile acids)^10^, this has not been explored using a global profiling technique such as NMR. ^1^H NMR spectroscopy detects protons within small molecules and produces a spectrum related to proton profile within the biofluid, thereby having particular utility as a global metabolic profiling technique, including host- and microbe-derived metabolites^11^. Other attractions of ^1^H-NMR includes its high-throughput nature, its reproducibility, and that it is non-destructive to the samples analysed. Data have been published regarding optimised faecal collection and preparation for ^1^H-NMR analysis^12^; such data have confirmed the detection of a range of gut microbial metabolites of key interest to health and disease, and which give potential insight into gut microbiome-host interactions. As an example, ^1^H-NMR typically allows the detection of short chain fatty acids (SCFAs)^12^, metabolites important for gut health, with strong links to microbial metabolism of polysaccharides^13^. However, although metabolic profiling from swabs has been shown to be effective and achievable in vaginal swabs^14^, data are lacking for the detection of metabolites from rectal swabs.

In this study, we extend previous work comparing gut microbiota composition between paired faecal and swab samples to also explore the comparability of gut microbiota functionality, using both Tax4Fun2, a tool to infer microbial functionality using 16S rRNA sequencing data and ^1^H-NMR spectroscopy-based metabolic profiling as our main tool of investigation.

## Methods

### Study design and sample collection

The research and all associated experimental protocols were performed in accordance with institutional approval from the Research Governance and Integrity Team of Imperial College London, London, UK, and ethical approval from a UK Research Ethics Committee (18/EM/0195; IRAS ref: 243310). Informed consent was obtained from all participants, and methods were carried out in accordance with relevant guidelines and regulations.

Matched faecal and rectal swab samples were obtained from 10 healthy individuals in a single centre in London, UK. All participants gave informed consent to take part. All participants took no regular medication, were non-smokers, and had not used antibiotics for at least 6 weeks prior to donation. Whole faeces were collected in a faeces collector (FECOTAINER®, AT Medical BV, The Netherlands) and COPAN FLOQSwabs™ (Copan Italia S.P.A., Brescia, Italy) were utilised as rectal swabs, given their previously demonstrated utility in faecal microbiota analysis^15^. The rectal swabs used were sterile with no preservative. Rectal swab collection was carried out at the same time as stool sample production and was obtained by self-insertion via the anus to a depth of 2–3 cm and rotated 3 times. Faecal samples and rectal swabs were stored at − 80 °C as crude samples without the use of any cryopreservative until processed. All samples were collected prior to December 2019, and as such, it is assumed that all participants were naïve to COVID-19 infection. Order of sampling was random based upon participant availability rather than a set order.

### 16S rRNA gene sequencing

DNA was extracted from crude faecal and swab samples using the DNeasy PowerLyzer PowerSoil Kit (Qiagen, Hilden, Germany) following manufacturer’s instruction with the modification that samples were homogenised in a Bullet Blender Storm bead beater (Chembio, St Alban’s, UK). DNA was quantified using a Qubit Fluorometer (ThermoFischer, UK), and was aliquoted and stored at − 80 °C until ready for downstream use. Sample libraries were prepared following Illumina’s 16S Metagenomic Sequencing Library Preparation Protocol^16^ using specifically designed V1/V2 hypervariable region primers^17^. Pooled sample library sequencing was performed using the Illumina MiSeq platform (Illumina Inc, Saffron Walden, UK) and the MiSeq Reagent Kit v3 (Illumina) using paired-end 300-bp chemistry. Processing of sequencing data was performed via the DADA2 pipeline (v1.18) as previously described^18^, using the SILVA bacterial database Version 138 (https://www.arb-silva.de/ (accessed on 28th July 2020)). Raw data were filtered to remove samples with a sequencing depth of < 1000 reads; furthermore, data were filtered to remove taxa that were not present in at least 10% of samples, to remove rare taxa that cannot be distinguished from sequencing artefacts (across all samples sequenced, mean sequencing depth was 22,842 reads, with a standard deviation of ± 8060). In addition, 16S rRNA gene qPCR was performed to determine total bacterial biomass within each sample, using qPCR primers and protocol as previously described^19^, enabling transformation of compositional metataxonomic data into ecosystem abundance^20^, and removing the need for rarefaction^21^.

After filtering of raw sequences via DADA2, they were passed into Phangorn for the construction of the phylogenetic tree using the default settings^22^. A combination of R packages were used to analyse and visualise faecal/ swab microbiota sequencing data, including Phyloseq^23^, Vegan^24^, and ggplot2^25^. Comparison of faecal and rectal swab microbiome taxonomy and ecological metrics was performed in R-studio (V1.2.5042). Shannon’s diversity index, Inverse-Simpson’s, Chao1 richness and Faith’s phylogenetic diversity were used to calculate alpha-diversity; beta-diversity analysis was primarily assessed using Aitchison’s distance^26^ after center log-ratio data transformation (CLR)^20^ (CLR was performed using the zCompositions R package for imputation of left-censored data)^27^, as well as via unweighted UniFrac. Principal coordinates analyses (PCoA) were generated to visualise the (dis)similarity between treatments and a permutational multivariate analysis of variance (PERMANOVA) statistically compared groupings within the data^24,28^. Extended error bar plots of taxonomic data were generated using the Statistical Analysis of Metagenomic Profiles (STAMP) software package using two-sided White’s nonparametric t-test with Benjamini–Hochberg FDR^29,30^. In addition, to putatively predict microbial functions from 16S rRNA gene sequencing data, the software Tax4fun2 v1.1.5 was used, with predicted relative values for different KEGG orthologues obtained, using log transformed data^31^.

### Metabolomic profiling using ^1^H-NMR

#### Sample preparation and data acquisition

Faecal water (FW) extracts for both faecal and rectal swab samples were obtained and analysed in 3.0 mm NMR tubes, as per previously described protocols^12,32^, with the additional step of vortexing/sonicating swabs (described further in Supplementary Methods). Rectal swabs and faecal sample extracts were randomised and analysed using a Bruker 600 MHz AVANCE III NMR spectrometer at 300 K. The 1D ^1^H NMR spectra were acquired using a standard one-dimensional pulse sequence, with saturation of the water resonance (noesygppr1d pulse program) during both the relaxation delay (RD = 4 s) and mixing time (t_m_ = 10 ms). In total, 4 dummy scans, 128 scans and 64 K data points were collected. Further information regarding comprehensive NMR set up parameters for all spectra acquired can be found in the Supplementary Methods.

#### Metabolomic data processing

1D ^1^H-NMR spectra were processed using vendor software TopSpin v3.5 (Bruker) and were automatically phased, baseline corrected and referenced to TSP. Data were imported into MATLAB (2014a, MathWorks) and redundant spectral regions corresponding to residual water (δ^1^H 4.67–4.92), TSP (δ^1^H  − 0.5 to 0.85), and noise (δ^1^H 8.67–11.0) were removed. The spectra of swab blanks confirmed the presence of a poly(ethylene glycol) derivative, acetone, acetate, formate, ethanol, methanol, compounds related to plastics, and traces of lactate (Supplementary Table 2); therefore, their spectral regions were removed to ensure uniformity and reliability when comparing between the spectra of FW with those of swab samples. Data were normalised using probabilistic quotient normalisation (PQN) to compensate for differences in concentration^33^.

#### Identification of metabolites

Metabolite annotation was carried out using selective 1D TOCSY, 2D-NMR experiments, and correlation spectroscopy on 1D ^1^H-NMR data set^34^. Internal and external databases such as the Human Metabolome Data Base (HMDB; http://hmdb.ca/)^35^ and/or the Biological Magnetic Resonance Data Bank (BMRB; http://www.bmrb.wisc.edu) were used for confirmation of assignments.

#### Data analysis

Correlation between relative units in matched faecal and swab samples of different alpha diversity metrics, paired data for each KEGG orthologue (KO; in the case of Tax4Fun2 data), and log-transformed paired metabolite values (in the case of ^1^H-NMR data) were analysed by Pearson’s coefficient (two-tailed analysis with P < 0.05 as cut off for significance and Benjamini–Hochberg test as false discovery rate correction). Analysis was performed using GraphPad Prism version 9.1.2 (225) (GraphPad Software, San Diego, California USA). Given the different extraction protocols used for metabolite profiling of stool and swab samples, a Mantel test was also applied for comparison, using Pearson’s product-moment correlation on log-transformed metabolomic data.

## Results

### The gut microbiota composition of matched faecal samples and rectal swabs is closely comparable

We firstly compared the 16S rRNA gene sequencing profiles for matched faecal samples and rectal swabs, comparing them both in terms of ecological metrics (diversity, richness, etc.) and specific profiles at different taxonomic levels.

**Figure 1:**
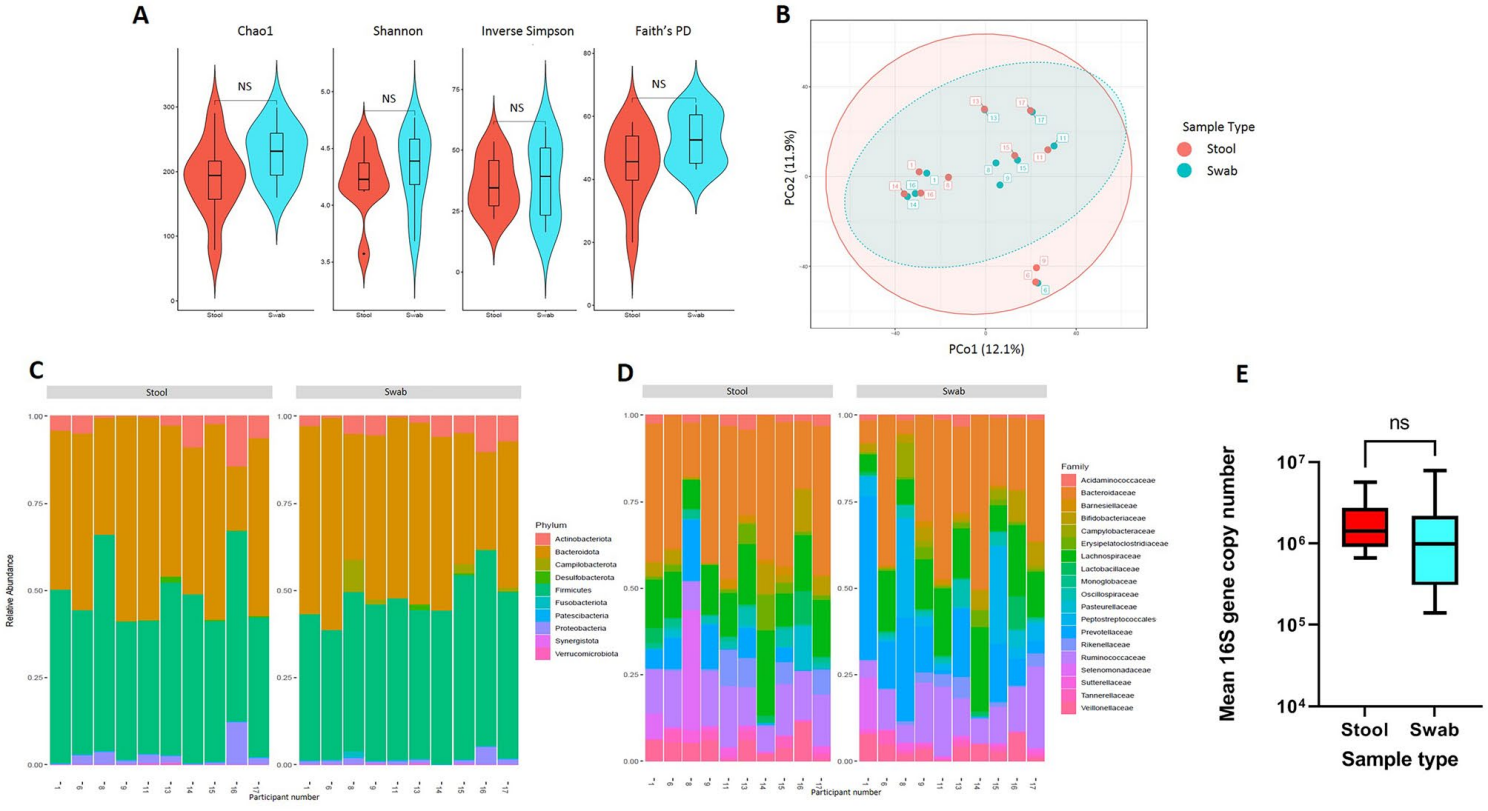
Comparison of compositional analysis of the gut microbiome, as assessed by matched faeces vs rectal swabs. (**A**) Alpha diversity metrics (Kruskal–Wallis). (**B**) Beta diversity, as represented by PCoA (numbers on points within figure represent study participant number). (**C**) Relative abundance plots of all bacterial phyla. (**D**) Relative abundance plot of major bacterial families (filtering used to remove families present at < 5% relative abundance; numbers on horizontal axis represent study participant number). (**E**) 16S rRNA gene copy number. Faeces: *n* = 10 samples; swabs: *n* = 10 samples.

The alpha (α)-diversity of all rectal swabs and faecal samples was analysed using a range of metrics; no statistically significant differences between values for swabs and faeces were found (*P* > 0.05, Kruskal–Wallis, Fig. 1A, Supplementary Fig. S1). Beta (β)-diversity showed expected inter-subject variability, but no statistically significant overall differences between rectal swabs and faecal samples when assessed using Aitchison’s distance after CLR (*P* = 0.982 PERMANOVA) (Fig. 1B); conversely, when comparing groups by unweighted UniFrac, no significant difference was seen in beta-dispersion (*P* = 0.9031) but was by PERMANOVA (*P* = 0.002); Supplementary Fig. 2. Of note, the major annotated bacterial phyla (including *Firmicutes, Bacteroidetes* and *Proteobacteria*) showed no statistically significant differences in relative abundance between swab and faecal samples (*q* > 0.05, White’s non-parametric two-sided t-test, Benjamini–Hochberg FDR correction; Fig. 1C). The only phylum showing significance between groups was *Campilobacterota*, which was enriched in swabs relative to faeces (*q* = 0.016; Supplementary Fig. 3A); however, this was only a feature in two participants, and made up < 10% of the overall reads in those participants. Similarly, only 5 out of 35 annotated bacterial families (Fig. 1D, Supplementary Fig. 3B), 9 out of 75 genera (Supplementary Fig. 3) and 10 out of 126 amplicon sequence variants (ASVs) demonstrated statistically significant differences in relative abundance between faecal samples and matched swabs; regarding bacterial families, it was noteworthy that no such differences were seen in the predominant families of *Bacteroidaceae, Lachnospiraceae, Prevotellaceae,* or *Ruminococcaceae.* Interestingly, 16S rRNA gene copy number in the DNA extracted from samples was not found to be different between stool and swab (*P* > 0.05, Mann–Whitney, Fig. 1E). When 16S rRNA gene sequencing relative abundance data from samples was corrected for bacterial biomass as derived from 16S rRNA gene qPCR^36^, there again remained close comparability of stool and swab microbiome compositional profiles, including for both the predominant and less prevalent bacteria at a particular taxonomic level (Supplementary Fig. 5). These data build upon the conclusions from other studies that a rectal swab is an appropriate substitute for a faecal sample for profiling of gut microbiome composition.

### Matched faecal samples and rectal swabs demonstrate comparable functionality, in terms of both inferred function and metabolome

**Figure 2:**
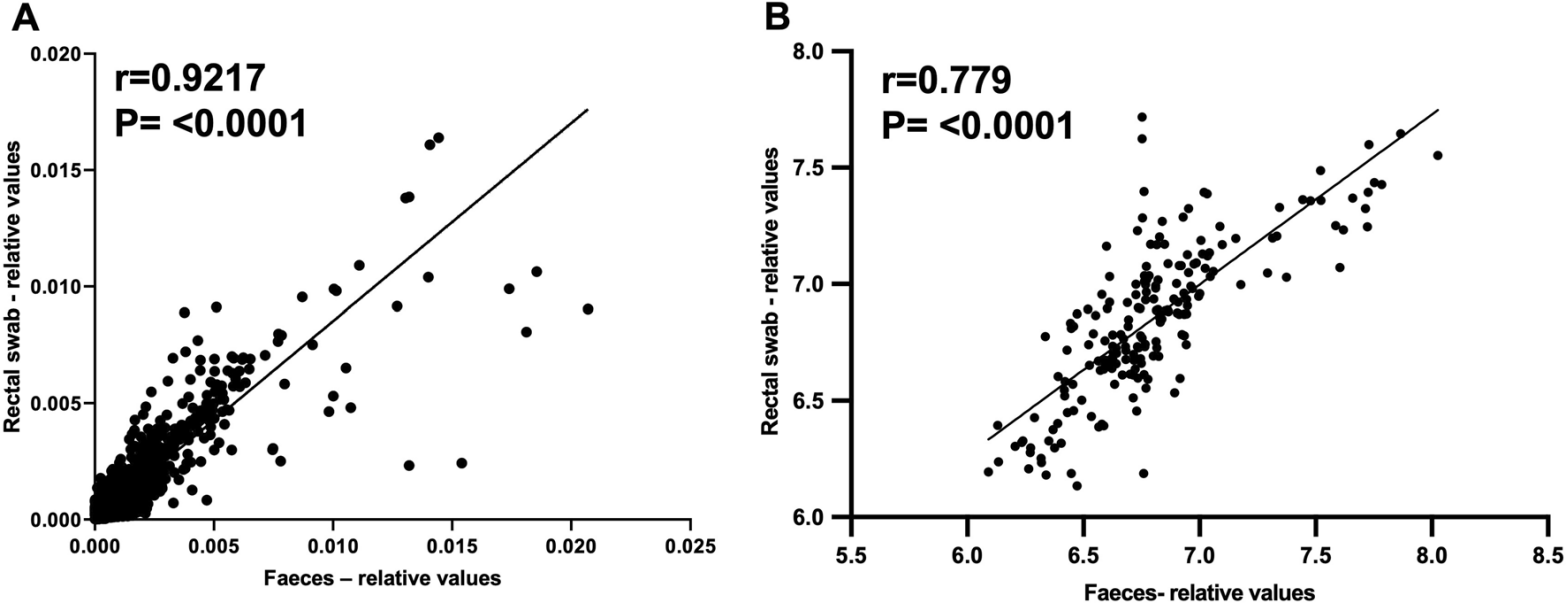
Comparison of gut microbial functionality in matched faeces vs rectal swabs. (**A**) Correlation of swab and faecal KEGG orthologue predicted gene abundance data obtained from Tax4Fun2, quantified as relative units (performed using Pearson’s coefficient); (**B**) correlation of all relative abundance values (log transformed and PQN normalised) for identified metabolites from faecal and swab samples (performed using Pearson’s coefficient). For (**A**), each dot represents paired stool and swab predicted gene abundance values for a particular KEGG orthologue for a particular sample; for (**B**), each dot represents paired stool and swab relative abundance values for a particular metabolite for a particular sample. Faeces: *n* = 10 samples; swabs: *n* = 10 samples.

Given our particular interest in gut microbiota functionality, we went on to compare inferred functionality (using predicted relative values of KEGG orthologues, derived using Tax4Fun2), as established from matched rectal swabs and faecal samples. We observed very close correlation and excellent comparability of the data obtained using both sampling techniques, as analysed by Pearson’s correlation (r = 0.9217, *P* < 0.0001; Fig. 2A). Predicted KO data were also CLR-transformed and compositionally tested (i.e. Euclidean distance) for groupwise differences between stool and swab; no statistical difference was observed (PERMANOVA, r2 value = 0.0258, P = 0.297), again consistent with comparable profiles in both sample types.

We next investigated the degree to which metabolomic profiles obtained from faecal samples and matched rectal swabs using ^1^H-NMR were similar. Firstly, we used established NMR protocols^32^ to identify and quantify a range of different metabolites from spectral profiles that could be reliably recognised in both groups of samples, focusing on gut microbial metabolites or those with an association to host-microbial interactions that are reliably identified by NMR^12^; 20 such metabolites were identified (Supplementary Table 2). On correlation of all relative abundances together between faecal and swab samples for these identified metabolites, excellent correlation was found (r = 0.7779, P < 0.0001, q = 0.0021, Benjamini–Hochberg false discovery rate correction (FDR); Fig. 2B). Conversely, the Mantel statistic (based on Pearson’s product-moment correlation) for stool and swab metabolites was r = 0.08767 (P = 0.294), suggesting that the different extraction protocols used between stool and swab samples did impact upon metabolic profiles observed, with dissimilarity between faecal and swab metabolomes.

**Figure 3:**
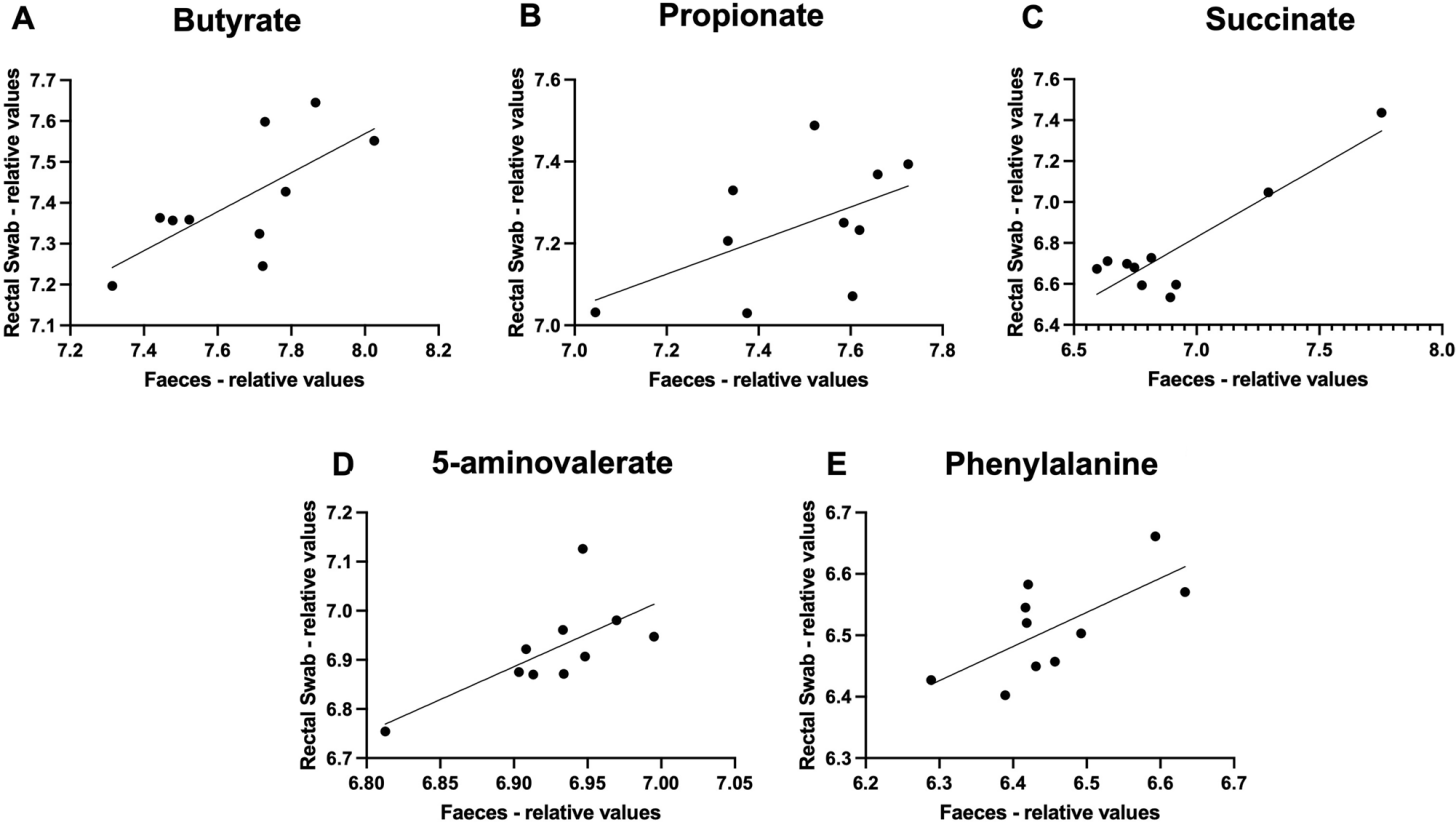
Correlation of levels of selected gut microbial-related metabolites in rectal swabs and matched stool samples. Performed using log transformed data with PQN normalisation. (**A**) Butyrate (*r* = 0.6945, *P* = 0.0258; q = 0.105); (**B**) propionate (*r* = 0.5298, *P* = 0.1152; q = 0.3456); (**C**) succinate (*r* = 0.8945, *P* = 0.0005; q = 0.00525); (**D**) 5-aminovalerate (*r* = 0.6816, *P* = 0.003; q = 0.105); (**E**) phenylalanine (*r* = 0.6877, *P* = 0.0279; q = 0.105). Faeces: *n* = 10 samples; swabs: *n* = 10 samples.

Further univariate analysis was performed on metabolites identified by ^1^H-NMR in both faeces and swabs, with a particular focus on those with a gut microbial origin. Specifically, we evaluated correlation between faecal and swab results for the identified short chain fatty acids; this analysis showed good correlation for butyrate (r = 0.6945, P = 0.0258, q = 0.105; Fig. 3A), but more modest correlation for propionate (r = 0.5298, P = 0.1152, q = 0.3456; Fig. 3B). Furthermore, good correlation was seen for several metabolites closely associated with gut microbiome-host interactions, including succinate (r = 0.8945, P = 0.0005, q = 0.0053; Fig. 3C), 5-aminovalerate (r = 0.6816, P = 0.003, q = 0.105; Fig. 3D), and phenylalanine (r = 0.6877, P = 0.0279, q = 0.105; Fig. 3E). However, the strength of correlation between swab and faecal data from other identified metabolites was more variable, and less strong overall (Supplementary Fig. 6). Of note, a general pattern was observed for those of the annotated metabolites with higher overall relative values (particularly in swabs) being those with the strongest correlation between rectal and stool values.

## Discussion

While colonic biopsies and faecal samples have a well-established role in profiling different aspects of the gut microbiota, both have drawbacks associated with their use. Clear attractions for the potential use of rectal swabs include the ease with which they can be administered and transported, and the high levels of acceptability to patients/research study participants^6^. Earlier studies which compared rectal swabs to colonic biopsy samples did not demonstrate favourable correlation between the microbiota from the two communities^7,37,38^; however, mucosal microbiota samples in the studies were obtained after bowel purgatives with the authors acknowledging this limitation^7^. Additionally, rectal swabs may not obtain the same mucosal adherent microbiota as biopsy samples, which may explain the poor correlation between the sampling types^39^. Conversely, the same publications^7,38^ and recent work^1,8,40–45^ have demonstrated rectal swab microbiota communities to be closely related to matched faecal samples. Although there is heterogeneity between studies in terms of sample storage, populations sampled (healthy controls or disease) and microbial analysis techniques, studies tend to demonstrate with overall consistency that rectal swabs are a reliable proxy of faecal sampling for microbiota compositional analyses. Our data add to a body of evidence demonstrating comparability of microbiota profiles between stool and swab samples, with only very modest differences in taxonomic composition observed. Of the few bacteria identified as differing significantly in abundance between sample type, we were not able to find an obvious unifying biological factor (e.g. sensitivity to oxygen, taxonomic relationship, etc.) that might link them. While a concern related to bacterial contamination may exist related to rectal swabs (given the manipulation required for their use), we reassuringly did not see any overrepresentation of skin-related bacteria (including streptococci and staphylococci) in rectal swabs relative to stool.

To date, previous publications using rectal swabs in gut microbiota research have mainly focused on the composition of the bacterial community, with data lacking with regards to the functionality of the microbiota and host interaction^1,7,8,37,38,40–45^. One study linked swab microbiota populations to gut microbiota functionality by interpreting KEGG pathways^38^, but generally there is a paucity of data with regards to profiling of microbiota functionality with rectal swabs. More specifically, there is a particular lack of data exploring the relationship between rectal swabs and stool for the assessment of other ‘omic’ profiles related to the microbiota, including the gut metabolome. In the current study, we used ^1^H-NMR (as a means of global metabolite profiling) to investigate this area, with our particular focus on metabolites related to host-microbiota interactions, given the growing interest of gut microbial metabolites to health and disease states, with one particular example of the latter being inflammatory bowel disease (IBD)^46^. The SCFAs butyrate and propionate are understood to be relevant to the pathogenesis of IBD, with previous work illustrating levels of these metabolites to be closely correlated to populations of *Faecalibacterium prausnitzii* and *Roseburia hominis,* bacteria which are known to be less abundant in active inflammation^47^. *F. prausnitzii* itself is a microbe of interest in IBD with a higher abundance noted in responders compared with non-responders to biologic medication^48^. Moreover, SCFAs themselves are thought to exert direct anti-inflammatory effects, such as inhibition of the pro-inflammatory cytokine tumour necrosis factor alpha (TNF-α) production from neutrophils^49^. Our research indicates that rectal swabs sample these SCFAs at comparable levels to corresponding faecal samples. Interestingly, our work also noted acceptable levels of correlation between both sample types in levels of other metabolites relevant to microbiome research, including succinate (a metabolite which has been implicated in fistulizing Crohn’s disease (CD)^50^ and is an important substrate to improve glucose homeostasis^51^), 5-aminovalerate (associated with proline metabolism pathways^52^), and phenylalanine (an amino acid found to be enriched in the gut in IBD^53^). However, more variable levels of correlation were found for other annotated metabolites, particularly for those identified at lower relative values. One potential explanation for this is what may be expected intuitively regarding swab use, i.e., that biomass of material obtained by swab sampling may be a factor that influences the metabolite profile that may be obtained. Options to mitigate this issue may include using alternative swab designs that may facilitate collection of material, and/or the use of more than one swab per collection; however, as ever, such options must be balanced against acceptability to patients, one of the major drivers towards consideration of swab use in the first place. Another possible explanation for any disparity between rectal and swab metabolic profiles may also represent the practicalities of sample handling. More specifically, previous work from our laboratory observed that, for ^1^H-NMR analysis of a faecal sample to be fully representative, the whole sample requires homogenisation, to account for differences in metabolic profile on the surface *versus* within the stool, likely reflecting oxygen exposure and its impact upon stool microbe metabolism, and freezing within 4–6 h, both of which may be cumbersome. By their nature^12^, rectal swabs require no initial sample handling phase and are easy to freeze, so may give a more representative simple ‘snapshot’ of the gut metabonome. Newer, reliable methods of stool sampling exist such as OMNIgene-GUT®, which has good results in microbial DNA analyses^54^; further recent work demonstrates good correlation between selected bile acids as detected in crude stool and via collection using OMNIgene-GUT®, but with a significantly reduced concentration of total bile acids using this kit compared to faecal sampling^10^.

Whilst our results are promising, our study does have limitations, and further work would be required before utilizing rectal swabs more broadly as a tool to study the gut metabolome instead of stool. We recruited a relatively small number of participants, and trends of the abundance of some metabolites (including propionate) may have been significant in a larger population; reassuringly, the faecal microbiome and metabolome profiles that we observed in our healthy participants showed close comparability to those described in larger scale studies of healthy populations^55^. Swabs were self-administered, and we do not know if different participants used the swab differently (e.g. depth of anorectal insertion) despite the standardised instructions for administration that they received; this could have affected the amount of material obtained and in turn influenced results. However, one of the key goals of exploring use of rectal swabs was to consider if these may be suitable for use by patients in clinic and trial settings without the need for healthcare professional direction; as such, we used self-administration in our protocol to mirror the envisaged ‘real world’ use of swabs. Our collection from healthy individuals allowed for snap freezing and prompt storage of samples, which may not always be feasible if swabs are utilised in a clinical outpatient setting. Of interest, even though overall metabolite correlation between swabs and faeces was very good, certain individual metabolites did not demonstrate such strong correlation; whether this represents the limit of detection of ^1^H-NMR, the volatility of particular metabolites, or other factors requires further exploration. In addition, while use of ^1^H-NMR for faecal metabolite profiling has a number of advantages for use in studies such as this (as discussed in the Introduction), it does not allow us to robustly assess a number of key metabolite groups associated with gut microbiome functionality, including bile acids and indole/tryptophan-related metabolites; use of other high sensitivity metabolomics pipelines—including mass spectrometry techniques—may be more appropriate for particular metabolite groups. Overall, it can be inferred that certain metabolites may be less detectable by rectal swabs, but more data in a larger population are required.

## Conclusion

While several early studies in different settings have suggested that rectal swabs may have utility in identifying gut microbiota composition comparable to faecal samples, the data regarding their use as a tool in identifying the gut metabolome remain more limited. This question is particularly pertinent given the growing role of omic studies—including those focused on gut microbial metabolites—as a route to exploring gut microbiome-host interactions. In this study, we use ^1^H-NMR (as a global metabolic profiling modality) to demonstrate that rectal swabs show promise as a tool to analyse both the gut microbial functionality (including the metabolome) and bacterial compositional profile with comparable efficacy to faecal samples, but that further method development is required before they might be suitable for more widespread clinical adaptation.

## Supplementary Information

Supplementary Information.

## Data Availability

Sequencing data from this study (in fastq-format) are publicly available for download at the European Nucleotide Archive (ENA) database using study accession number PRJEB50814 (http://www.ebi.ac.uk/ena/data/view/PRJEB50814). Other datasets used and/or analysed during the current study (i.e. ^1^H-NMR) are available from the corresponding author on reasonable request.

## References

[1] Williams GM (2019). Gut microbiome analysis by post: Evaluation of the optimal method to collect stool samples from infants within a national cohort study. PLoS ONE.

[2] Lecky DM, Hawking MK, McNulty CA (2014). Patients' perspectives on providing a stool sample to their GP: A qualitative study. Br. J. Gen. Pract..

[3] Marechal C (2017). Compliance with the faecal calprotectin test in patients with inflammatory bowel disease. United Eur. Gastroenterol. J..

[4] Jalanka J (2015). Effects of bowel cleansing on the intestinal microbiota. Gut.

[5] HPS. *Toolkit for the Early Detection, Management and Control of Carbapenemase-Producing Enterobacteriaceae in Scottish Acute Settings* (2016).

[6] Currie K (2018). The acceptability of screening for carbapenemase producing enterobacteriaceae (CPE): Cross-sectional survey of nursing staff and the general publics' perceptions. Antimicrob. Resist. Infect. Control.

[7] Budding AE (2014). Rectal swabs for analysis of the intestinal microbiota. PLoS ONE.

[8] Reyman M, van Houten MA, Arp K, Sanders EAM, Bogaert D (2019). Rectal swabs are a reliable proxy for faecal samples in infant gut microbiota research based on 16S-rRNA sequencing. Sci. Rep..

[9] Lamichhane S, Sen P, Dickens AM, Oresic M, Bertram HC (2018). Gut metabolome meets microbiome: A methodological perspective to understand the relationship between host and microbe. Methods.

[10] Neuberger-Castillo L, Ammerlaan W, Betsou F (2021). Fitness for purpose of stabilized stool samples for bile acid metabolite analyses. Sci. Rep..

[11] Nicholson JK, Lindon JC (2008). Metabonomics. Nature.

[12] Gratton J (2016). An optimized sample handling strategy for metabolic profiling of human feces. Anal. Chem..

[13] Miller TL, Wolin MJ (1996). Pathways of acetate, propionate, and butyrate formation by the human fecal microbial flora. Appl. Environ. Microbiol..

[14] Pruski P (2017). Medical swab analysis using desorption electrospray ionization mass spectrometry: A noninvasive approach for mucosal diagnostics. Anal. Chem..

[15] Tedjo DI (2015). The effect of sampling and storage on the fecal microbiota composition in healthy and diseased subjects. PLoS ONE.

[16] Amplicon, P., Clean‐Up, P. & Index, P. *16s Metagenomic Sequencing Library Preparation*. www.Illumina.com (2013).

[17] Mullish BH (2018). Functional microbiomics: Evaluation of gut microbiota-bile acid metabolism interactions in health and disease. Methods.

[18] Callahan BJ (2016). DADA2: High-resolution sample inference from Illumina amplicon data. Nat. Methods.

[19] Mullish BH (2019). Microbial bile salt hydrolases mediate the efficacy of faecal microbiota transplant in the treatment of recurrent *Clostridioides difficile* infection. Gut.

[20] Gloor GB, Macklaim JM, Pawlowsky-Glahn V, Egozcue JJ (2017). Microbiome datasets are compositional: And this is not optional. Front. Microbiol..

[21] McMurdie PJ, Holmes S (2014). Waste not, want not: Why rarefying microbiome data is inadmissible. PLoS Comput. Biol..

[22] Schliep KP (2011). phangorn: Phylogenetic analysis in R. Bioinformatics.

[23] McMurdie PJ, Holmes S (2013). phyloseq: An R package for reproducible interactive analysis and graphics of microbiome census data. PLoS ONE.

[24] Oksanen, J. *et al. Package ‘Vegan’*. https://cran.r-project.org, https://github.com/vegandevs/vegan (2020).

[25] Wickham H (2016). ggplot2 Elegant Graphics for Data Analysis.

[26] Aitchinson. *The Statistical Analysis of Compositional Data J.R. Stat soc.pdf* (1982).

[27] Palarea-Albaladejo J, Martín-Fernández JA (2015). zCompositions—R package for multivariate imputation of left-censored data under a compositional approach. Chemom. Intell. Lab. Syst..

[28] Oksanen, J. *et al. Package ‘Vegan’ Community Ecology Package*. https://cran.r-project.org/web/packages/vegan/vegan.pdf (2022).

[29] Parks DH, Tyson GW, Hugenholtz P, Beiko RG (2014). STAMP: Statistical analysis of taxonomic and functional profiles. Bioinformatics.

[30] Benjamini Y, Hochberg Y (1995). Controlling the false discovery rate: A practical and powerful approach to multiple testing. J. R. Stat. Soc..

[31] Wemheuer F (2020). Tax4Fun2: Prediction of habitat-specific functional profiles and functional redundancy based on 16S rRNA gene sequences. Environ. Microbiome.

[32] Dona AC (2014). Precision high-throughput proton NMR spectroscopy of human urine, serum, and plasma for large-scale metabolic phenotyping. Anal. Chem..

[33] Dieterle F, Ross A, Schlotterbeck G, Senn H (2006). Probabilistic quotient normalization as robust method to account for dilution of complex biological mixtures. Application in 1H NMR metabonomics. Anal. Chem..

[34] Posma JM (2012). Subset optimization by reference matching (STORM): An optimized statistical approach for recovery of metabolic biomarker structural information from 1H NMR spectra of biofluids. Anal. Chem..

[35] Wishart DS (2018). HMDB 4.0: The human metabolome database for 2018. Nucleic Acids Res..

[36] Jian C, Luukkonen P, Yki-Jarvinen H, Salonen A, Korpela K (2020). Quantitative PCR provides a simple and accessible method for quantitative microbiota profiling. PLoS ONE.

[37] Araujo-Perez F (2012). Differences in microbial signatures between rectal mucosal biopsies and rectal swabs. Gut Microbes.

[38] Jones RB (2018). Inter-niche and inter-individual variation in gut microbial community assessment using stool, rectal swab, and mucosal samples. Sci. Rep..

[39] Shen TD (2021). The mucosally-adherent rectal microbiota contains features unique to alcohol-related cirrhosis. Gut Microbes.

[40] Bassis CM (2017). Comparison of stool versus rectal swab samples and storage conditions on bacterial community profiles. BMC Microbiol..

[41] Bokulich NA, Maldonado J, Kang DW, Krajmalnik-Brown R, Caporaso JG (2019). Rapidly processed stool swabs approximate stool microbiota profiles. mSphere..

[42] Biehl LM (2019). Usability of rectal swabs for microbiome sampling in a cohort study of hematological and oncological patients. PLoS ONE.

[43] Fair K (2019). Rectal swabs from critically ill patients provide discordant representations of the gut microbiome compared to stool samples. mSphere..

[44] Short MI (2021). Comparison of rectal swab, glove tip, and participant-collected stool techniques for gut microbiome sampling. BMC Microbiol..

[45] Mazzarelli A (2021). 16S rRNA gene sequencing of rectal swab in patients affected by COVID-19. PLoS ONE.

[46] Lloyd-Price J (2019). Multi-omics of the gut microbial ecosystem in inflammatory bowel diseases. Nature.

[47] Machiels K (2014). A decrease of the butyrate-producing species *Roseburia hominis* and *Faecalibacterium prausnitzii* defines dysbiosis in patients with ulcerative colitis. Gut.

[48] Radhakrishnan ST (2022). Systematic review: The association between the gut microbiota and medical therapies in inflammatory bowel disease. Aliment Pharmacol. Ther..

[49] Vinolo MA (2011). Suppressive effect of short-chain fatty acids on production of proinflammatory mediators by neutrophils. J. Nutr. Biochem..

[50] Ortiz-Masia D (2020). Succinate activates EMT in intestinal epithelial cells through SUCNR1: A novel protagonist in fistula development. Cells.

[51] De Vadder F (2016). Microbiota-produced succinate improves glucose homeostasis via intestinal gluconeogenesis. Cell Metab..

[52] Bisht V (2021). Integration of the microbiome, metabolome and transcriptomics data identified novel metabolic pathway regulation in colorectal cancer. Int. J. Mol. Sci..

[53] Bosch S (2018). Fecal amino acid analysis can discriminate de novo treatment-naive pediatric inflammatory bowel disease from controls. J. Pediatr. Gastroenterol. Nutr..

[54] Neuberger-Castillo L (2020). Method validation for extraction of DNA from human stool samples for downstream microbiome analysis. Biopreserv. Biobank.

[55] Zierer J (2018). The fecal metabolome as a functional readout of the gut microbiome. Nat. Genet..

