# Supplementary material for: Rectal swabs as a viable alternative to faecal sampling for the analysis of gut microbiota functionality and composition
Source: Sci Rep. 2023 Jan 10;13:493. doi: 10.1038/s41598-022-27131-9 (PMC9831010; doi:10.1038/s41598-022-27131-9)
Supplement: Supplementary file 1 — Supplementary Information. [file 41598_2022_27131_MOESM1_ESM.docx]

**Supplementary Data:**

**RECTAL SWABS AS A VIABLE ALTERNATIVE TO FAECAL SAMPLING FOR THE ANALYSIS OF GUT MICROBIOTA FUNCTIONALITY AND COMPOSITION**

1. **Supplementary Methods:**

**Sample preparation for ^1^H-NMR analysis**

Given the different nature of stool and swab samples, comparable but separate preparation protocols were used.

For stool samples, faecal water (FW) was made using two parts Ultra High Performance Liquid Chromatography water (UHPLC H_2_O) to one-part faeces in an Eppendorf tube, which was then vortexed for 5 minutes. These samples were then centrifuged at 20,000 *g* and 4C° for 20 minutes, and the supernatant transferred to a fresh Eppendorf tube with 1.5M KH_2_PO_4_ buffer (pH 7.4, 100% of deuterium oxide (D_2_O), 2 mM sodium azide, and 1% of TSP (3-trimethylsilyl-[2,2,3,3,-^2^H_4_]-propionic acid sodium salt) in a 9:1 ratio, respectively. This mixture was vortexed, briefly centrifuged, and the supernatant pipetted into the NMR tube.

Regarding rectal swabs, rectal swab sample tips were broken off at a pre-marked breakpoint approximately 2 cm from the swab tip into an Eppendorf tube. An aliquot of 300 μL of 1.5M KH_2_PO_4_ buffer-UHPLC H_2_O mixture (1:9 ratio) was added to the tube, and the samples were vortexed and sonicated for 5 min. These samples were then centrifuged at 20,000*g* and 4C° for 20 minutes. The swab tip was removed, samples were briefly centrifuged, and the supernatant was pipetted into NMR tubes.

For all prepared samples, a total volume of 180 μL was pipetted into 3 mm NMR tubes.

**^1^H-NMR set-up parameters:**

Rectal swabs and faecal sample extracts were analysed using a Bruker 600 MHz AVANCE III NMR spectrometer at precisely 300 K. The 1D ^1^H NMR spectra were acquired using a standard one-dimensional pulse sequence, with saturation of the water resonance (noesygppr1d pulse program) during both the relaxation delay (RD = 4s) and mixing time (t_m_ = 10 ms). For both sets of samples, a standard one-dimensional NOESY pulse sequence was acquired using a pulse width of approximately 13 µs. During this time, a water pre-saturation pulse was applied to suppress the water signal. The two magnetic field z-gradients implemented were applied for 1 ms, the receiver gain was set to 90.5 and acquisition time (ACQ) to 2.73s for all experiments. Each spectrum was acquired using 4 dummy scans, 128 scans, 64 K data points and a spectral window of 20 ppm. Prior to Fourier Transformation, each free induction decay was multiplied by an exponential function corresponding to a line broadening of 0.3 Hz.

2D ^1^H−^1^H *J*-resolved experiments were also acquired for each sample to detect the *J*-couplings in the second dimension using the pulse program with suppression of the water resonance during the relaxation delay (jresgpprqf). The parameters set for this pulse sequence were as follows: 16 dummy scans and 2 scans, 8K data points with a spectral window of 16.7 ppm for f2 and 40 increments with spectral window of 78 Hz for f1. Incremented delay of 3µs, RD of 2 s, and ACQ of 0.41 s. The receiver gain was set to 90.5. A sine-bell apodization function was applied on both dimensions, followed by Fourier transformation, tilting by 45°, and symmetrisation along f1^26^.

Selective 1D TOCSY sequence and 2D NMR experiments with water pre-saturation during relaxation delay including gradient ^1^H–^1^H TOtal COrrelation SpectroscopY (TOCSY) and ^1^H–^13^C Heteronuclear Single Quantum Coherence (HSQC) were acquired from a representative swab sample for identification purposes.

1. **Supplementary Tables:**

| **Participant Characteristics** | **Healthy Volunteers** | | |  |
| --- | --- | --- | --- | --- |
|  |  |  |  |  |
| **Sex** | Male |  | 8 |  |
|  | Female |  | 2 |  |
| **Age (mean + range)** |  | 31 (24 - 36) | |  |
|  |  |  |  |  |
| **Ethnicity** | White British |  | 4 |  |
|  | White Spanish |  | 1 |  |
|  | British Indian |  | 4 |  |
|  | British Sri Lankan |  | 1 |  |

**Supplementary Table 1: Demographic details of study participants**

| **Name** | **Assignment** | | | | | | | | | | **Confirmation Experiments** |
| --- | --- | --- | --- | --- | --- | --- | --- | --- | --- | --- | --- |
| Butyrate | **0.89 (t)*** | 1.56 (sext) | 2.16 (t) |  |  |  |  |  |  |  | 1D, JRES, TOCSY, HSQC |
| Isoleucine | 0.94 (t) | **1.01 (d)** | 1.26 (m) | 1.47 (m) | 1.99 (m) |  |  |  |  |  | 1D, JRES, TOCSY, HSQC |
| Leucine | **0.96 (d)** | 0.97 (d) | 1.70 (m) | 1.74 (m) |  |  |  |  |  |  | 1D, JRES, TOCSY, HSQC |
| Valine | **0.99 (d)** | 1.05 (d) | 2.28 (m) | 3.62 (d) |  |  |  |  |  |  | 1D, JRES, TOCSY, HSQC |
| Propionate | **1.06 (t)** | 2.19 (q) |  |  |  |  |  |  |  |  | 1D, JRES, TOCSY, HSQC |
| Lactate | **1.33 (d)** | 4.12 (q) |  |  |  |  |  |  |  |  | 1D, JRES, TOCSY, HSQC |
| Threonine | 1.34 (d) | 3.6 (d) | **4.26 (m)** |  |  |  |  |  |  |  | 1D, JRES, TOCSY, HSQC |
| Lysine | 1.46 (m) | **1.72 (m)** | 1.91 (m) | 3.03 (t) | 3.77 (t) |  |  |  |  |  | 1D, sel-TOCSY, JRES, TOCSY, HSQC |
| Alanine | **1.48 (d)** | 3.79 (q) |  |  |  |  |  |  |  |  | 1D, JRES, TOCSY, HSQC |
| 5-aminovalerate | 1.63 (m) | 1.65 (m) | **2.24 (t)** | 3.02 (t) |  |  |  |  |  |  | 1D, sel-TOCSY, JRES, TOCSY, HSQC |
| Glutamate | 2.06 (m) | **2.14 (m)** | 2.36 (m) | 3.77 (dd) |  |  |  |  |  |  | 1D, JRES, TOCSY, HSQC |
| Methionine | 2.14 (m) | 2.14 (s) | 2.21 (m) | **2.65 (t)** | 3.87 (m) |  |  |  |  |  | 1D, JRES, TOCSY |
| Succinate | **2.41 (s)** |  |  |  |  |  |  |  |  |  | 1D, JRES, HSQC |
| 3-hydroxyphenylpropionate | 2.47 (t) | 2.84 (t) | 6.75 (m) | 6.81 (m) | **6.87 (m)** | 7.25 (t) |  |  |  |  | 1D, sel-TOCSY, JRES, TOCSY, HSQC |
| Aspartate | 2.69 (dd) | **2.82 (dd)** | 3.91 (dd) |  |  |  |  |  |  |  | 1D, JRES, TOCSY, HSQC |
| Trimethylamine | **2.88 (s)** |  |  |  |  |  |  |  |  |  | 1D, JRES |
| Tyrosine | **3.06 (dd)** | 3.2 (dd) | 6.9 (d) | 7.19 (d) |  |  |  |  |  |  | 1D, JRES, TOCSY, HSQC |
| Phenylalanine | **3.13 (d)** | 3.29 (dd) | 7.33 (m) | 7.38 (m) | 7.43 (m) |  |  |  |  |  | 1D, JRES, TOCSY, HSQC |
| a/b-Glucose | **3.25 (dd)** | 3.41 (m) | 3.42 (m) | 3.50 (m) | 3.54 (m) | 3.72 (m) | 3.85 (m) | 3.9 (m) | 4.65 (d) | 5.24 (d) | 1D, JRES, TOCSY, HSQC |
| Serine | 3.85 (dd) | 3.96 (dd) | **4.00 (dd)** |  |  |  |  |  |  |  | 1D, JRES, TOCSY, HSQC |

**Supplementary Table 2: Details of ^1^H-NMR assignment strategy**

1. **Supplementary Figures:**

**Supplementary Figure 1: Pearson’s correlation of alpha diversity metrics between stool and swab.** A) Chao 1; B) inverse Simpson; C) Shannon; D) Faith’s PD. Faeces: *n*=10 samples; swabs: *n*=10 samples.

**
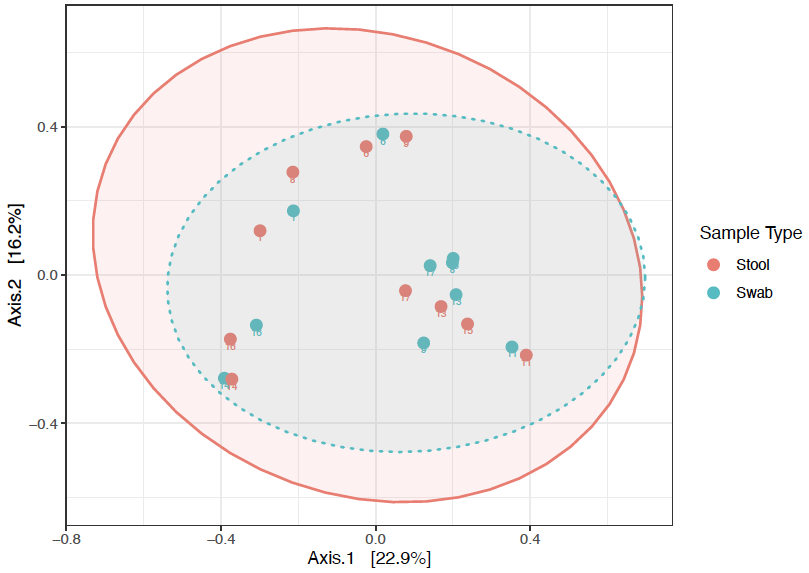
**

**Supplementary Figure 2: Unweighted UniFrac assessment of beta-diversity for stool and swab samples.** As represented by PCoA. Faeces: *n*=10 samples; swabs: *n*=10 samples.

**Supplementary Figure 3: Extended error bar plot of differences in relative abundance of bacteria between faecal and matched swab samples at key taxonomic levels.** A) Bacterial phyla; B) Bacterial families. Statistically assessed using White’s non-parametric two-sided t-test with Benjamini-Hochberg false discovery rate correction. Plots are ordered by effect size; only *q* values < 0.1 displayed. Faeces: *n*=10 samples; swabs: *n*=10 samples. SV = sequence variants.


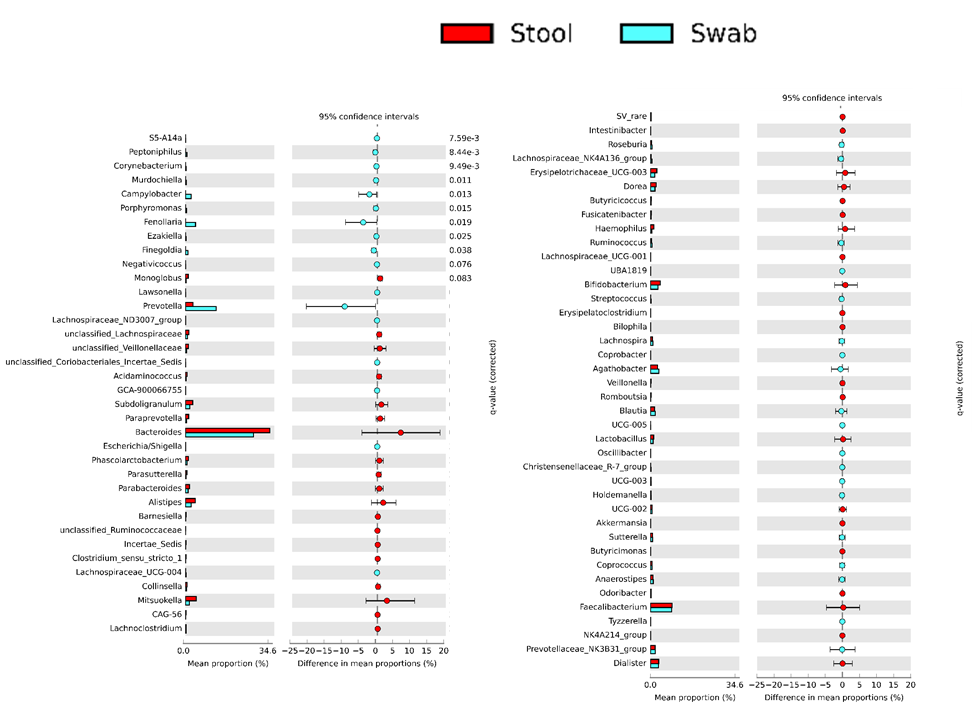


**Supplementary Figure 4: Extended error bar plot of differences in relative abundance of bacteria between faecal and matched swab samples at genus level.** Statistically assessed using White’s non-parametric two-sided t-test with Benjamini-Hochberg false discovery rate correction. Plots are ordered by effect size; only *q* values < 0.1 displayed. Faeces: *n*=10 samples; swabs: *n*=10 samples. SV = sequence variants.

**Supplementary Figure 5: Phylum-level 16S rRNA gene profiling of stool and swabs, corrected for bacterial biomass:** Bacterial biomass as established using 16S qPCR. Only phyla present in at least 25% of samples are included. Statistically assessed using Mann-Whitney test. NS=non-significant. Faeces: *n*=10 samples; swabs: *n*=10 samples.

**Supplementary Figure 6: Correlation of levels of other annotated gut microbial-related metabolites in rectal swabs and matched stool samples.** A) aspartate; B) methionine; C) lactate; D) glutamate; E) tyrosine; F) a/b-glucose; G) 3-hydroxyphenylpropionate; H) alanine; I) isoleucine; J) leucine; K) lysine; L) serine; M) threonine; N) trimethylamine; O) valine. Faeces: *n*=10 samples; swabs: *n*=10 samples. PQN = probability quotient normalisation.
